# A bibliometric and visualized in oral microbiota and cancer research from 2013 to 2022

**DOI:** 10.1007/s12672-024-00878-5

**Published:** 2024-02-01

**Authors:** Zhiyu Gu, Yunkun Liu

**Affiliations:** Hospital of Stomatology, Zunyi Medical University, Zunyi, 563000 China

**Keywords:** Oral microbiota, Cancer, Bibliometric analysis, VOSviewer, WoSCC

## Abstract

Numerous studies have highlighted the implication of oral microbiota in various cancers. However, no bibliometric analysis has been conducted on the relationship between oral microbiota and cancer. This bibliometric analysis aimed to identify the research hotspots in oral microbiota and cancer research, as well as predict future research trends. The literature published relating to oral microbiota and cancer was searched from the Web of Science Core Collection database (WoSCC) from 2013 to 2022. VOSviewer or Citespace software was used to perform the bibliometric analysis, focusing on countries, institutions, authors, journals, keywords and references. A total of 1516 publications were included in the analysis. The number of publications related oral microbiota and cancer increased annually, reaching its peak in 2022 with 287 papers. The United States (456) and China (370) were the countries with the most publications and made significant contributions to the field. Sears CL and Zhou XD were the most productive authors. The high frequency of keywords revealed key topics, including cancer (colorectal cancer, oral cancer), oral microbiota (*Fusobacterium nucleatum*, *Porphyromonas gingivalis*), and inflammation (periodontal disease). The latest trend keywords were *F. nucleatum*, dysbiosis, prognosis, tumor microenvironment, gastric microbiota, complications and survival, suggesting a new hotspot in the field of oral microbiota and cancer. Our study provides a comprehensive analysis of oral microbiota and cancer research, revealing an increase in publications in recent years. Future research directions will continue to focus on the diversity of oral microbiota impacted by cancers and the underlying mechanism connecting them, providing new ideas for targeted therapy of tumorigenesis.

**Supplementary Information:**

The online version contains supplementary material available at 10.1007/s12672-024-00878-5.

## Introduction

Cancer, known as the most disturbing human disease and the second leading cause of death worldwide, encompasses several types including lung, liver, prostate, colorectal, stomach, breast, and oral cancers [1, 2]. It can be caused by various factors, including heritable and environmental risk factors [3]. One such environmental factors is microorganisms, which have a long-term coexistence with the human body. In 2022, Douglas Hanahan proposed polymorphic microbiomes as one of the hallmarks of cancer, suggesting that microorganisms symbiotically associated with the body can profoundly impact cancer development, progression, and response to therapy [4, 5]. The International Agency for Cancer Research has identified 11 microorganisms, such as *Helicobacter pylori*, hepatitis B virus and hepatitis C virus, as human carcinogens [6]. For instance, there is a wealth of evidence suggesting that bacteria-mediated inflammation, like *Helicobacter pylori*, is linked to the development of gastric cancer and colorectal cancer in humans [7, 8].

The oral microbiota is composed of various microbes found in the oral cavity, including more than 770 bacterial species, such as bacteria, fungi, viruses, and bacteriophages. The primary genera include *Streptococcus*, *Haemophilus*, *Leptotrichia*, *Porphyromonas*, *Prevotella*, *Propionibacterium*, *Staphylococcus*, *Veillonella*, and *Treponema* [9]. It is considered the second greatest reservoir of microbiota in the human body, after the gut, and plays a crucial role in maintaining health [10, 11]. However, plenty of studies have showed that when the ecological balance is disrupted, potential opportunistic pathogens can overgrow, leading to oral and systemic diseases, such as dental caries, periodontitis, peri-implantitis, mucosal diseases, oral cancer, rheumatoid arthritis, diabetes, aspiration pneumonia, osteomyelitis, inflammatory bowel disease, and cardiovascular disease [12–17].

Advancements in sequencing and mass spectrometry technologies have facilitated extensive microbial profiling of various cancers by analyzing the microbiota of dental plaque (calculus), saliva, oral rinse, or tissues [18–21]. Disease-related oral microbiota are involved in cancers and has long been studied, such as *Porphyromonas gingivalis* and *Fusobacterium nucleatum* [22, 23]. Furthermore, there is substantial evidence indicating that oral microbiota is implicated in numerous cancers in distant organs, including colorectal cancer, liver cancer, lung cancer, throat cancer, esophageal cancer, oropharyngeal cancer, pancreatic cancer, genitourinary cancer, gastric cancer, leukaemia, and hematological cancer [24–33]. The alteration of inflammation within the microenvironment and interference with host cell signaling pathways involved in cellular proliferation, differentiation, and viability by oral microbiota can provide insights into the mechanisms of tumorigenesis [34]. Understanding these mechanisms could contributes to supportive care and precise treatment of tumors [35, 36].

Bibliometric analysis is a mathematical and statistical method used to evaluate literature information in a specific research interest. It utilizes tools like VOSviewer or Citespace to identify key authors, institutions, countries, important journals, hotspots and emerging trends. These analyses provide rich visualization information that helps explore the knowledge domain and enhance understanding of research activity [37]. Recently, several bibliometric analyses have been conducted on the oral microbiome. For instance, Liao Ga et al.(2020) performed a global analysis evaluated trends and hotspots in oral microbiome research, suggests focusing on early childhood caries, squamous cell carcinoma, gut microbiome, *Helicobacter pylori*, *Candida albicans*, and dysbiosis [38]. Li Zhengrui et al. also conducted a decade-long bibliometric analysis (2013–2022) on oral microbiome research, providing a comprehensive understanding of its role in systemic diseases and conditions. This study contributed to the development of novel therapies and preventive strategies for improving human oral health and overall well-being [39]. Additionally, Sa’ed H Zyoud et al. (2022) conducted a visualization analysis on the links between the gut microbiome and cancer, revealing that ‘microbiota composition and gene expression’ and ‘host-microbiome interaction in cancer immunotherapy’ would remain research hotspots [40]. Despite numerous reviews on oral microbiota and tumors, no bibliometric analysis has been performed or published this topic [3, 9, 41]. Therefore, this study aims to comprehensively analyze the research situation and trends in oral microbiota and cancer-related literature, with the goal of improving our understanding of cancer development and management of cancer outcomes.

## Material and methods

### Data sources and collection

The Web of Science Core Collection (WoSCC) database is a widely used bibliometric analysis database that indexes research data sets from over 33,000 journals. To ensure database stability, we retrieved literature from the WoSCC database from January 1, 2013, to December 31, 2022. The Science Citation Index Expanded served as the data source. We employed the following search terms in our topics: ‘cancers’, ‘neoplasm’, ‘tumors’, ‘oral microbiota’, ‘salivary microorganism’, ‘subgingival plaque microbiota’, ‘subgingival plaque microorganism’, ‘mucosal microbiota’, ‘mouth microbiota’, and ‘dental microbiota’. The detailed search strategy is presented in the supplementary materials. Following the search strategy, we focused on articles and reviews published in English to exclude irrelevant content and ensure the research results' integrity and accuracy. We downloaded the ‘Full Record and Cited References’ of these research papers from the WoSCC database, converted them to.txt format, and imported them into VOSviewer or CiteSpace for visualization analysis. We also downloaded the WoSCC database analysis results for further analysis.

### Data analysis and visualization

We utilized VOSviewer 1.6.14 to conduct an analysis and visualization of the data. Our analysis included examining the co-occurrence of keywords to identify research trends and hotspots. Additionally, we investigated research collaborations through the analysis of citations, including authors, organizations, countries, and sources. To ensure representativeness, we set certain parameters. For keywords, we considered those that occurred at least 15 times, resulting in 146 keywords meeting the threshold out of a total of 7059 keywords. Similarly, we considered authors affiliated with at least 5 different countries, resulting in 45 countries meeting the threshold. We also analyzed 2248 organizations, with a minimum threshold of 5, and found 163 organizations meeting the criteria. Furthermore, we examined 9458 authors, with a minimum threshold of 5, and identified 43 authors meeting the criteria. In terms of co-cited authors, we analyzed 53,425 cases, with a minimum threshold of 25, and found 247 authors meeting the criteria. Additionally, we examined 642 published journals, with a minimum threshold of 5, and identified 61 journals meeting the criteria. Lastly, we analyzed 8518 co-cited journals, with a minimum threshold of 70, and found 249 journals meeting the criteria. It is important to note that our analysis includes the United Kingdom, which encompasses England, Northern Ireland, Scotland, and Wales. Similarly, our analysis includes Taiwan as part of China.

CiteSpace 6.2.6, a bibliometric tool, was utilized to analyze and visualize the co-occurrence of countries/regions, institutions, trends of high-frequency bursts keywords, and co-cited references. Duplicate literature was removed from the full list before the analysis. The specific parameters used were as follows: ‘Time slicing’ was set to 2013–2022 with a year per slice of 1. ‘Pruning’ was performed using ‘pruning sliced networks’ and ‘pathfinder’. The k value for g-index was adjusted to ensure that the size of the network does not exceed the version limit of 300. Other settings remained at their default values.

Microsoft Office Excel 2019 was used to manage all the data related to the articles included in the study. GraphPad Prism 8.0 was utilized to create histograms for annual publications, document types, funding agencies, research area, and publishers. The journal citation reports (JCR) quartiles of journals and 2022 impact factors (IF) were obtained from the most recent edition of JCR as of July 28, 2023. No statistical tests were performed, and no P values were determined..

## Results

### General information

**Fig. 1:**
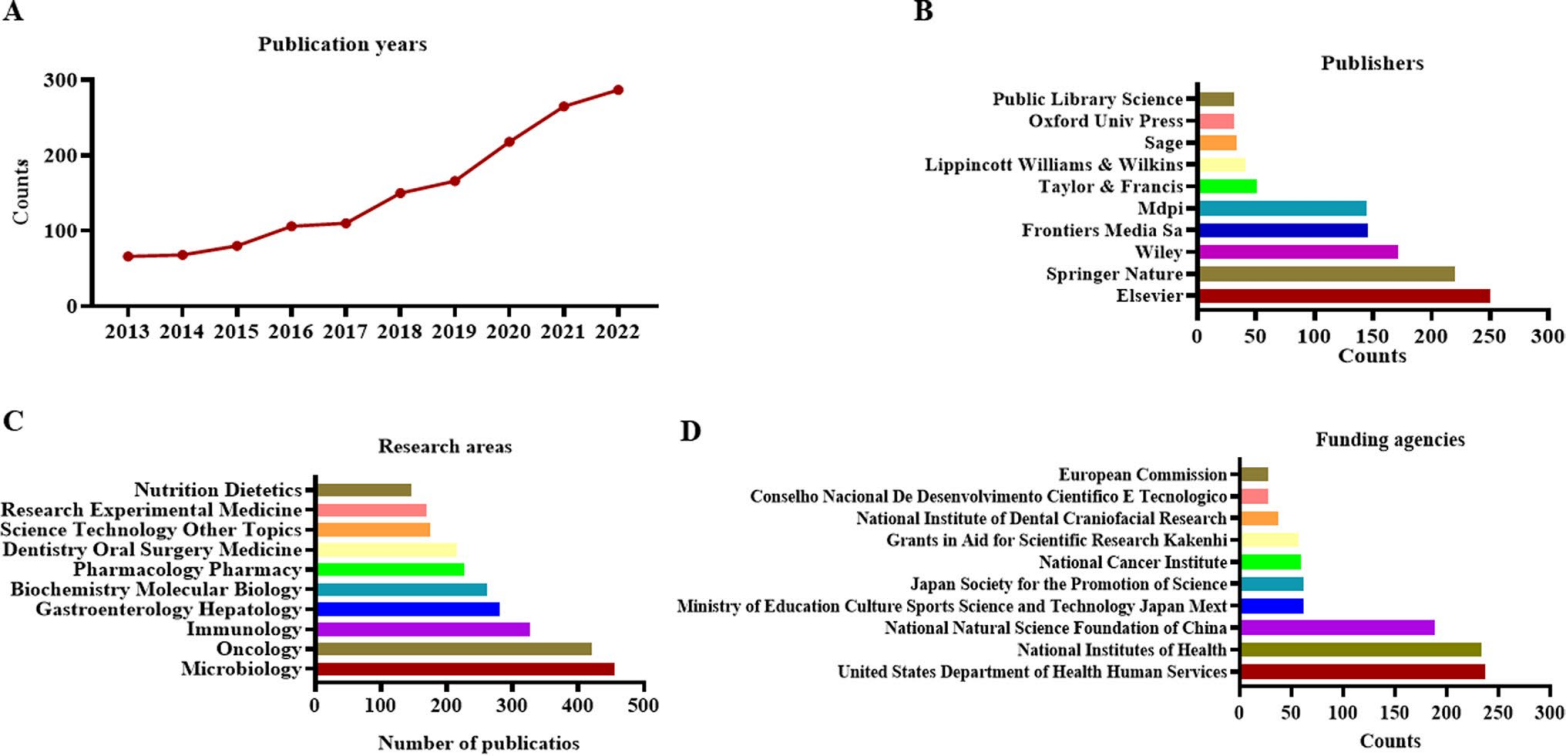
Annual publications (**A**), the top 10 publishers (**B**), the top 10 research areas (**C**) and the top 10 funding agencies and **D** in oral microbiota and cancer research

We collected a total of 1516 publications related to oral microbiota and cancer from the WoSCC database using our search strategies. These articles were contributed by researchers from 86 countries/regions, 2248 institutions, and 9458 authors, and were published in 642 different journals. Figure 1A illustrates a consistent increase in the number of publications on oral microbiota and cancer from 2013 to 2021, with an average of 151.6 articles published each year. The year 2022 recorded the highest number of publications (n = 287), followed by 2021 (n = 265) and 2020 (n = 218). Among the publishers, Elsevier had the most significant influence with 250 publications, followed by Springer Nature (n = 220) and Wiley (n = 172) (Fig. 1B). Figure 1C presents the top 10 research areas in oral microbiota and cancer research, with microbiology (n = 245), oncology (n = 227), dentistry oral surgery medicine (n = 202), immunology (n = 165), and biochemistry molecular biology (n = 112) being the most extensively studied areas. These findings can serve as a valuable reference for researchers intending to submit their research in this field.

### Countries/regions, institutions and funding agencies

**Fig. 2:**
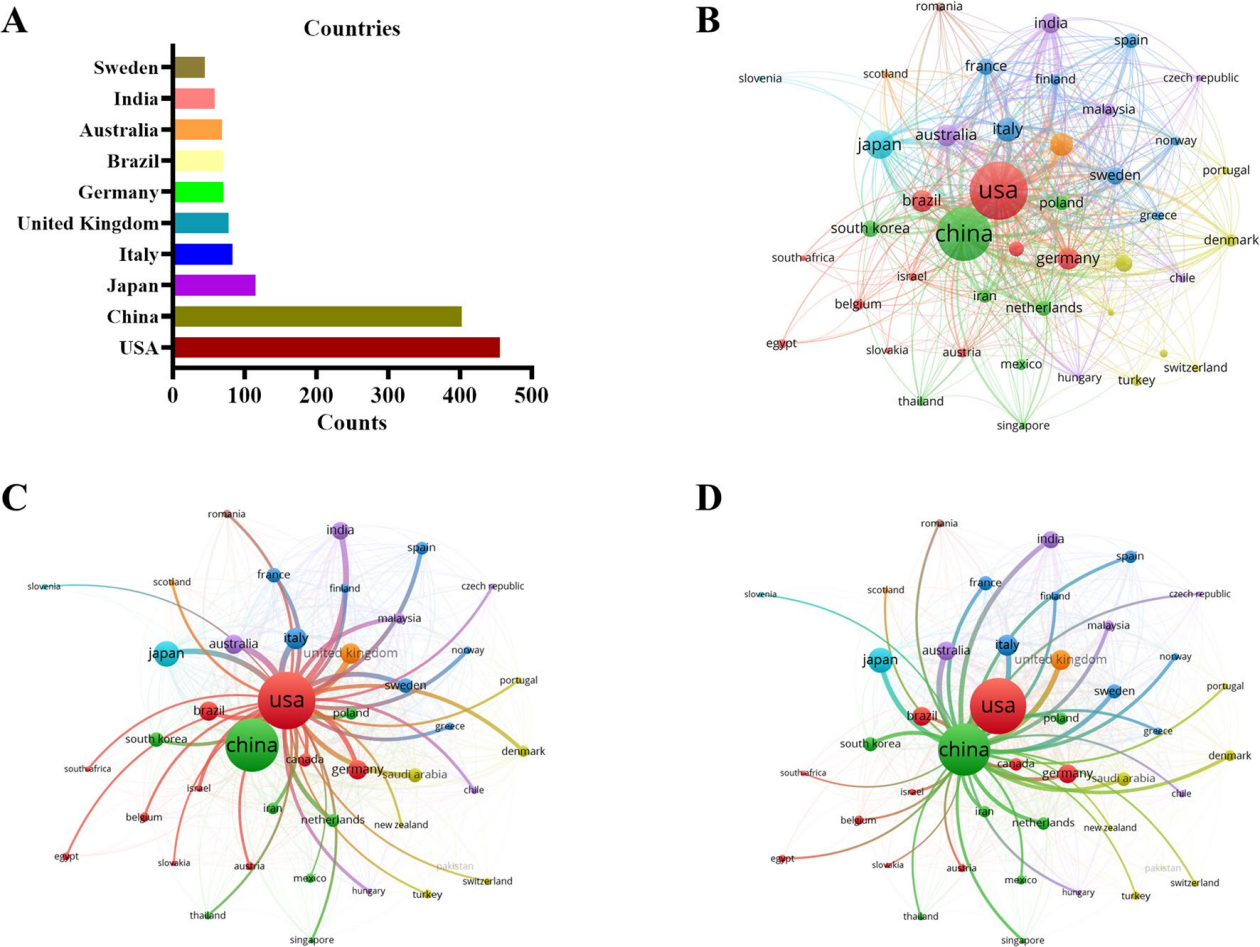
Articles related to the oral microbiota and cancer published by countries/regions. **A** The top 10 countries/regions with high publications in oral microbiota and cancer research. **B–D** The co-occurrence map of countries/regions

From 2013 to 2022, a total of 86 countries/regions conducted research on oral microbiota and its relation to cancer. As depicted in Fig. 2A, the USA emerged as the most productive country with 456 articles, followed by China with 402 articles. The combined contribution of these two countries accounted for 56.6% of the total output, amounting to 858 papers. Figure 2B indicates a strong connection between these countries, with a total link strength of 6501. The USA (Fig. 2C) and China (Fig. 2D, Fig. S1) collaborated with 41 other countries, with respective total link strengths of 2994 and 2393. Notably, there were active collaborations between the USA and China. Research funding plays a crucial role in shaping research directions, as evident from the top 10 funding agencies displayed in Fig. 1D. The United States Department of Health Human Services (n = 237) provided the highest support for publications, followed by the National Institutes of Health (n = 233) and the National Natural Science Foundation of China (n = 188). These findings highlight the significant funding contribution of the USA and China in this field.

**Fig. 3:**
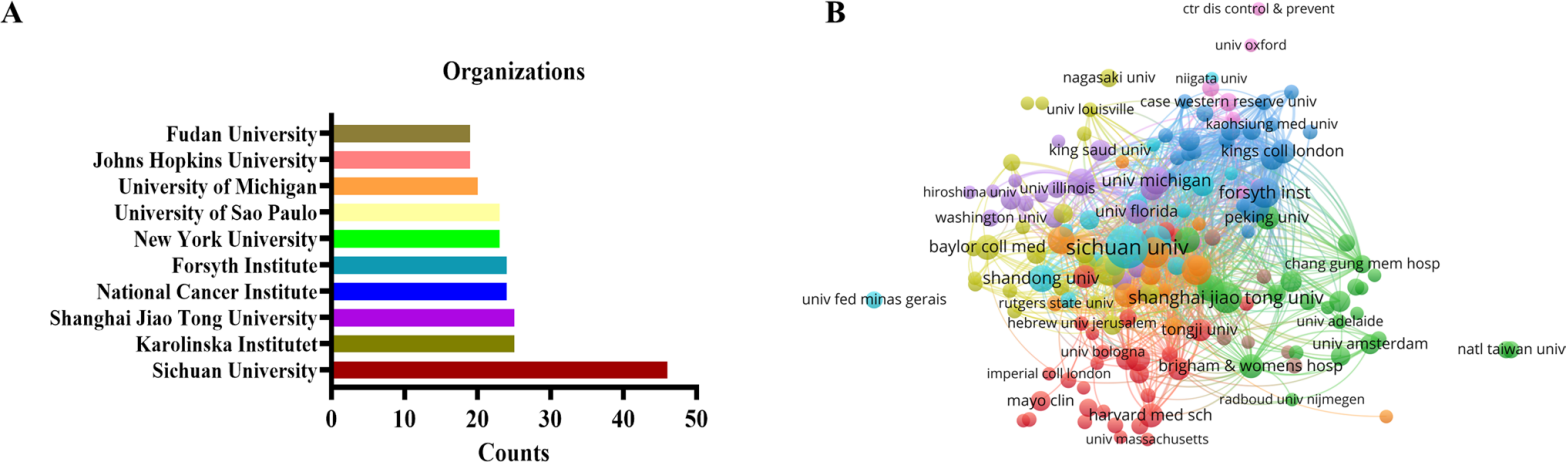
Articles related to the oral microbiota and cancer published by organizations. **A** The top 10 organizations with high publications in oral microbiota and cancer research. **B** The co-occurrence map of organizations

Among the top 10 institutions, three are from China, collectively contributing 90 publications. Sichuan University leads with 46 papers (996 citations) and 109 links, with a total link strength of 384. Shanghai Jiao Tong University published 25 papers (662 citations) with 94 links and a total link strength of 239. Fudan University published 19 papers, indicating China’s dominant role in the field of oral microbiota and cancer. Additionally, the USA published a total of 111 papers, solidifying its position as a research hub for oral microbiota and cancer research (Fig. 3, Fig. S2).

### Source journal analysis

**Fig. 4:**
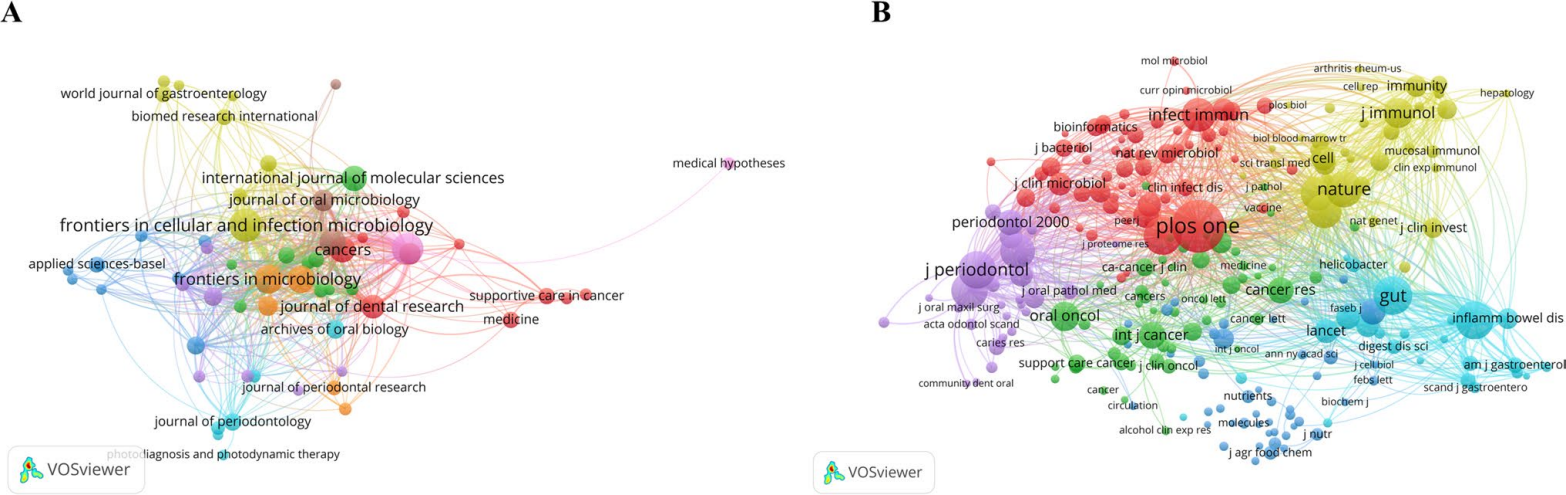
The co-occurrence map of journals (**A**) and co-cited journals (**B**) in oral microbiota and cancer research

A total of 1516 publications were analyzed in this study, which were published in 642 journals. Among these journals, 23 had more than 10 publications and 8 had more than 20 publications. The top 10 journals accounted for 279 articles, making up 18.4% of all articles. Five of these journals were from Switzerland, three from the UK, and two from the USA (Table 1, Fig. 4A). The most productive journal, *Frontiers in Cellular and Infection Microbiology*, published 42 articles with 43 links and a total link strength of 280. It was followed by *Scientific Reports* (n = 39) and *Frontiers in Microbiology* (n = 31). The impact factor (IF) of the top 10 journals ranged from 3.7 to 7.6, with the *Journal of Dental Research* having the highest IF (7.6). Most of these journals are in the Q2 division, indicating their potential for growth.

**Table 1:** Top 10 journals ranked by the number of papers published

Rank	Journal	N	IF(2022)	JCR quartiles	Country
1	Frontiers in Cellular and Infection Microbiology	42	5.7	Q2	Switzerland
2	Scientific Reports	39	4. 6	Q3	UK
3	Frontiers in Microbiology	31	5.2	Q2	Switzerland
4	Frontiers in Immunology	29	7.3	Q2	Switzerland
5	Plos One	27	3.7	Q3	USA
6	Cancers	26	5.2	Q2	Switzerland
7	International Journal of Molecular Sciences	25	5.6	Q2	Switzerland
8	Journal of Dental Research	22	7.6	Q1	USA
9	Journal of Oral microbiology	19	4.5	Q2	UK
10	Oral Diseases	19	3.8	Q3	UK

A network map was created using 249 cite-journals with at least 70 citations (Fig. 4B). Table 2 presents the top 10 cite-journals, with *Plos One* having the most citations (n = 2308), 248 links, and a total link strength of 134,182. It was followed by *Gut* (n = 1351) and *Scientific Reports* (n = 1319). The IF of the 10 cite-journals ranged from 3.7 to 64.8. Four journals had an IF of more than 20, namely *Nature* (IF = 64.8), *Science* (IF = 56.9), *Gastroenterology* (IF = 29.4), and *Gut* (IF = 24.5).

**Table 2:** The top 10 cite-journals with the most citations

Rank	Journal	Citations	IF(2022)	JCR quartiles	Country
1	Plos One	2308	3.7	Q3	USA
2	Gut	1351	24.5	Q1	UK
3	Scientific Reports	1319	4.6	Q3	UK
4	Gastroenterology	1294	29.4	Q1	UK
5	Journal of Periodontology	1266	4.3	Q2	USA
6	Journal of Clinical Periodontology	1219	6.7	Q1	UK
7	Nature	1217	64.8	Q1	UK
8	Journal of Dental Research	1112	7.6	Q1	USA
9	PNAS	1079	11.1	Q1	USA
10	Science	1078	56.9	Q1	USA

### Author analysis

**Fig. 5:**
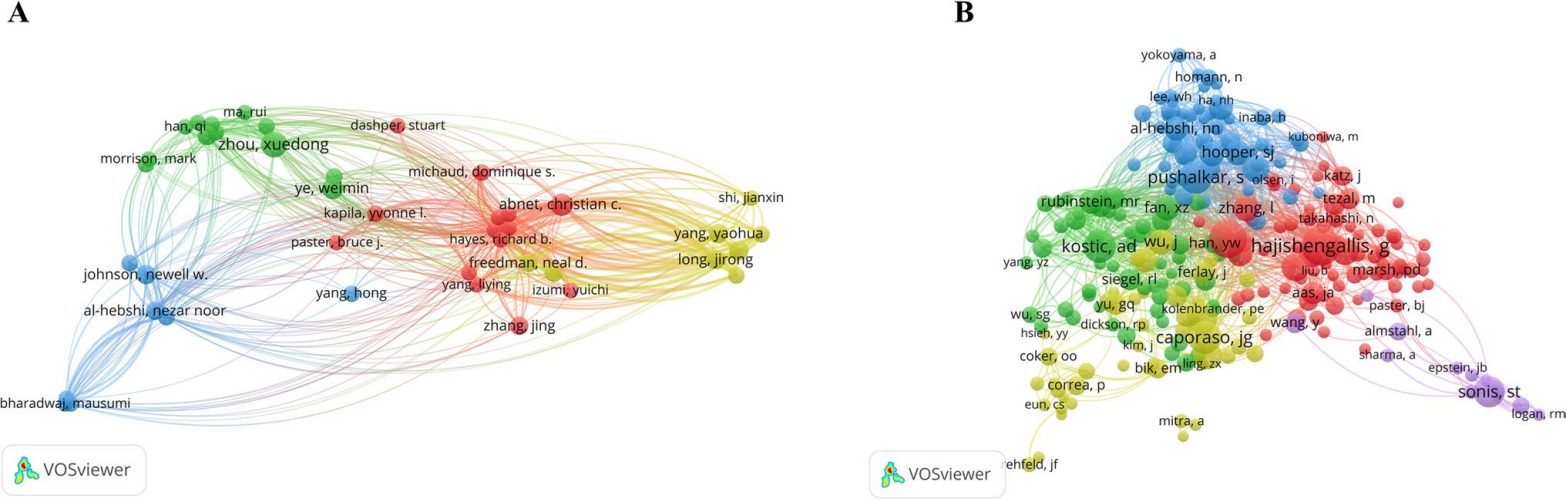
The co-occurrence map of authors (**A**) and co-cited authors (**B**) in oral microbiota and cancer research

This study examined a total of 9000 authors who have published articles in the field of oral microbiota and cancer. Among the top 10 authors, Sears CL (n = 15, 1378 citations), Zhou XD (n = 14, 272 citations), and Liu Y (n = 13, 137 citations) were found to be the most productive authors (Table 3). The network map included 43 authors who have published at least 5 papers. Notably, Zhou XD, Cheng L, Xu X, Peng X, Li JY, Ren B, Han Q, and Ma R, who are a team from Sichuan University, were clustered together in green color. Their research primarily focuses on the relationship between oral microbiota and oral diseases as well as systemic diseases. Additionally, they have established an oral microbiome sample bank for the Chinese population, which contributes to a better understanding of oral microbiota (Fig. S3). Another cluster in red color consists of Sears CL, Yu J, Abnet CC, and Petrosino JF, who actively collaborate in oral microbiota and cancer research (Fig. 5A).

**Table 3:** Top 10 authors and co-cited authors ranked by article counts

Rank	Author	Counts	Citations	Rank	Co-cited author	Citations
1	Sears CL	15	1378	1	Abnet CC	1297
2	Zhou XD	14	272	2	Ahn JY	817
3	Liu Y	13	137	3	Gapstur SM	817
4	Yu J	12	1417	4	Hayes RB	817
5	Li Y	12	268	5	Peters BA	817
6	Abnet CC	10	1297	6	Purdue MP	817
7	Li Y	10	207	7	Pei ZH	526
8	Li LJ	9	376	8	Yang LY	500
9	Ye WM	9	344	9	Freedman ND	483
10	Petrosino JF	9	338	10	Ye WM	344

Out of the 53,425 co-cited authors, 10 were co-cited over 300 times (Table 3). Abnet CC ranked first with 1297 citations, followed by Ahn JY (n = 817), Gapstur SM (n = 817), and Hayes RB (n = 817). The network map (Fig. 5B) included 247 co-cited authors who have published at least 25 papers, resulting in a total of 19,492 links and a total link strength of 97,335. In this network, Abnet CC, Freedman ND, and Ye WM were clustered together, indicating their close collaboration. Similarly, Ahn JY, Gapstur SM, Hayes RB, Peters BA, Purdue MP, Pei ZH, and Yang LY were clustered together, suggesting extensive contacts and cooperation among these authors.

### Keywords co-occurrence analysis

**Fig. 6:**
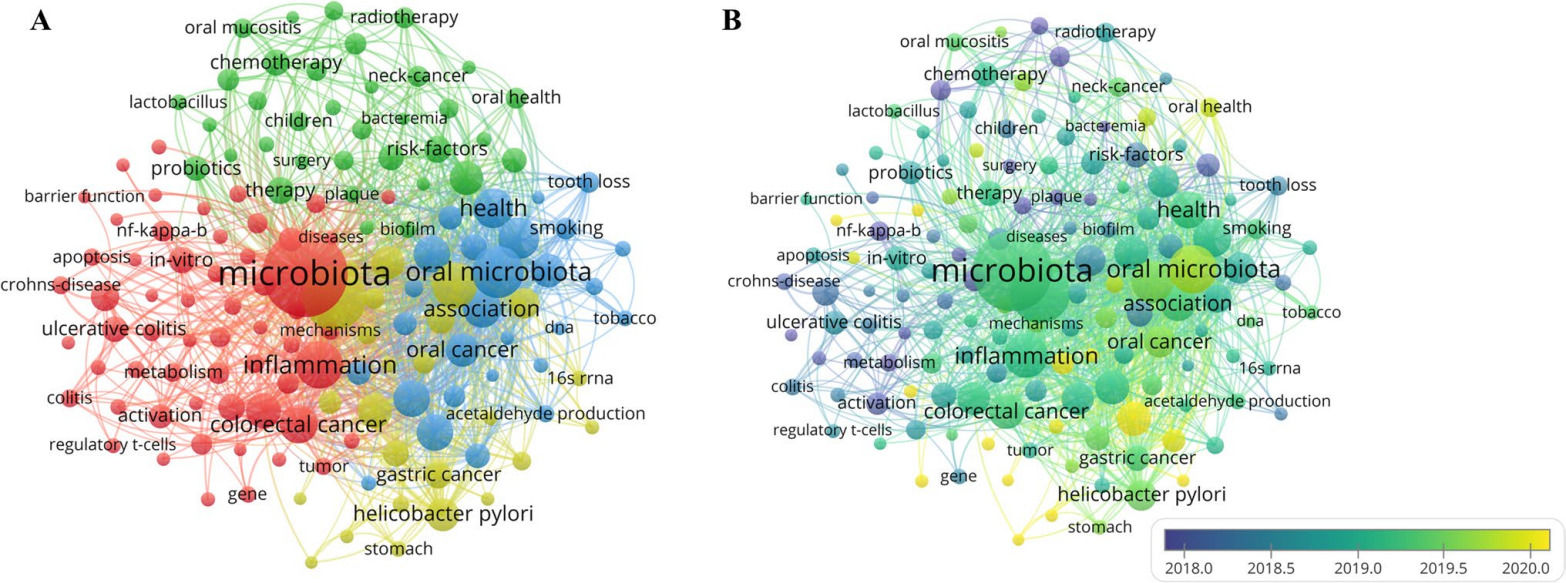
Keywords co-occurrence network (**A**) and overlay (**B**) visualization map in oral microbiota and cancer research

A total of 7059 keywords were identified in 1516 studies related to oral microbiota and cancer. A co-occurrence network and overlay visualization map were created using 146 keywords, resulting in four clusters displayed in different colors. The network consisted of 4952 links, with a total link strength of 16,654 (Fig. 6A). In cluster 1 (red), the prominent keywords included microbiota, inflammation, colorectal cancer, and expression. Cluster 2 (green) highlighted keywords such as head, therapy, risk-factors, chemotherapy, and dental caries. Cluster 3 (blue) showcased commonly mentioned keywords such as oral microbiota, periodontal disease, health, association, *Porphyromonas gingivalis*, periodontitis, *Fusobacterium nucleatum*, oral cancer, and squamous-cell carcinoma. Cluster 4 (yellow) encompassed keywords like cancer, risk, infection, disease, *Helicobacter pylori*, and saliva.

The top 20 keywords in oral microbiota and cancer research are presented in Table 4. Microbiota appeared most frequently with a count of 557, followed by cancer (n = 315), oral microbiota (n = 214), inflammation (n = 181), periodontal disease (n = 136), *P. gingivalis* (n = 112), and *F. nucleatum* (n = 109). *P. gingivalis*, *F. nucleatum*, and *Helicobacter pylori* represent different subtypes of oral microbiota. Oral cancer and colorectal cancer are representative tumor types. Inflammation and infection are potential pathogenic mechanisms.

**Table 4:** The top 20 keywords with the most citation count

Rank	Keyword	Count	Rank	Keyword	Count
1	Microbiota	557	11	*Porphyromonas gingivalis*	112
2	Cancer	315	12	*Fusobacterium nucleatum*	109
3	Oral microbiota	214	13	Expression	101
4	Inflammation	181	14	Infection	100
5	Risk	161	15	Oral cancer	94
6	Periodontal disease	136	16	Squamous cell carcinoma	94
7	Health	129	17	Head	93
8	Colorectal cancer	120	18	Disease	92
9	Association	118	19	*Helicobacter pylori*	90
10	Periodontitis	112	20	Saliva	74

Furthermore, Fig. 6B illustrates the annual distribution of keywords, highlighting the latest trends such as prognosis, dysbiosis, tumor microenvironment, gastric microbiota, and F. nucleatum, with an average publication year of 2020. Table 5 displays the top 43 keywords with the strongest citation bursts, which include prevalence, *Helicobacter pylori*, carcinogenesis, pancreatic cancer, oral squamous cell carcinoma, complications, and survival, particularly after 2019. These findings suggest a new area of interest in the field of oral microbiota and cancer.

**Table 5:** Top 43 keywords with the strongest citation bursts

Keywords	Year	Strength	Begin	End	2013—2022
Tumor necrosis factor	2013	6.69	2013	2014	
Dental implants	2013	6.12	2013	2015	
Activation	2013	5.78	2013	2016	
Oral bacteria	2013	5.29	2013	2016	
Mucositis	2013	3.78	2013	2014	
Cancer patients	2013	3.77	2013	2016	
Crevicular fluid	2013	3.15	2013	2014	
Care	2013	2.92	2013	2015	
Crohns-disease	2013	2.71	2013	2016	
Ulcerative colitis	2013	2.56	2013	2014	
Colon	2013	2.52	2013	2014	
Inflammatory bowel disease	2013	2.41	2013	2014	
Prevention	2014	4.8	2014	2017	
Microflora	2014	4.67	2014	2018	
Disease	2013	4.36	2014	2015	
Tooth loss	2014	3.41	2014	2017	
Risk factors	2014	3.27	2014	2017	
Oral hygiene	2014	2.91	2014	2017	
Bacteria present	2014	2.43	2014	2017	
Cancer-patients	2014	2.43	2014	2017	
Metabolism	2015	5.54	2015	2018	
Dental plaque	2013	5.4	2015	2016	
Double-blind	2015	4.73	2015	2018	
Aggressive periodontitis	2015	2.55	2015	2018	
Squamous cell carcinoma	2013	4.32	2016	2017	
In-vitro	2016	4.18	2016	2018	
Mortality	2016	3.53	2016	2019	
Responses	2016	3.29	2016	2018	
Mice	2016	2.89	2016	2017	
Risk-factors	2017	5.42	2017	2018	
Esophageal cancer	2017	3.11	2017	2020	
Tumor	2017	3.07	2017	2018	
Diagnosis	2018	3.61	2018	2020	
Prevalence	2016	3.64	2019	2020	
Women	2019	3.42	2019	2020	
Helicobacter-pylori	2019	3.42	2019	2020	
Carcinogenesis	2017	2.45	2019	2020	
System	2020	3.26	2020	2022	
Epithelial-cells	2018	2.99	2020	2022	
Pancreatic-cancer	2020	2.9	2020	2022	
Oral squamous cell carcinoma	2018	2.55	2020	2022	
Complications	2020	2.54	2020	2022	
Survival	2020	2.45	2020	2022	

### Citation and co-citation references

We utilized VOSViewer to identify the cited documents and co-cited references in studies related to oral microbiota and cancer. In Table 6, we presented the top 10 research articles based on their total citations, which reflects their scientific influence. These articles were cited a total of 5299 times during the study period, with an average citation rate of 529.9. It is noteworthy that 7 of these papers explicitly discussed the relationship between microbiota and cancer. Specifically, Fan XZ and Flemer B conducted studies on oral microbiota in relation to pancreatic and colon cancer, respectively.

**Table 6:** The top 10 citation analysis of cited reference

Rank	Author	Title	Journal	Year	Citations
1	Sivan A	Commensal Bifidobacterium promotes antitumor immunity and facilitates anti–PD-L1 efficacy	Science	2015	2034
2	O'keefe SJD	Fat, fibre and cancer risk in African Americans and rural Africans	Nature Communication	2015	560
3	Dejea CM	Microbiota organization is a distinct feature of proximal colorectal cancers	PNAS	2014	413
4	Jin CC	Commensal Microbiota Promote Lung Cancer Development via gd T Cells	Cell	2019	386
5	Brennan CA	Fusobacterium nucleatum-symbiont,opportunist and oncobacterium	Nature Reviews Microbiology	2019	374
6	Fan XZ	Human oral microbiome and prospective risk for pancreatic cancer: a population-based nested case–control study	Gut	2018	373
7	Boleij A	The Bacteroides fragilis Toxin Gene Is Prevalent in the Colon Mucosa of Colorectal Cancer Patients	Clinical Infectious Diseases	2015	315
8	Han YW	Mobile Microbiome: Oral Bacteria in Extra-oral Infections and Inflammation	Journal of Dental Research	2013	293
9	Coker OO	Mucosal microbiome dysbiosis in gastric carcinogenesis	Gut	2018	284
10	Flemer B	The oral microbiota in colorectal cancer is distinctive and predictive	Gut	2018	267

Out of the 74,709 co-cited references we retrieved, Table 7 presents the top 10 co-cited references. The most frequently co-cited reference was a review titled ‘The human oral microbiome’ published in the Journal of Bacteriology by Dewhirst FE, et al. in 2010. This was followed by an article titled ‘QIIME allows analysis of high-throughput community sequencing data’. We observed that these top 10 co-cited references can be categorized into three main themes: high-throughput sequencing technology, oral microbiota and oral cancer, and *F. nucleatum* and colorectal carcinoma.

**Table 7:** The top 10 co-citation analysis of cited reference

Rank	Author	Title	Journal	Year	Citations
1	Dewhirst FE	The Human Oral Microbiome	Journal of Bacteriology	2010	113
2	Caporaso JG	QIIME allows analysis of highthroughput community sequencing data	Nature Methods	2010	112
3	Segata N	Metagenomic biomarker discovery and explanation	Genome Biology	2011	94
4	Bray F	Global Cancer Statistics 2018: GLOBOCAN Estimates of Incidence and Mortality Worldwide for 36 Cancers in 185 Countries	CA: A Cancer Journal for Clinicians	2018	91
5	Schmidt BL	Changes in Abundance of Oral Microbiota Associated with Oral Cancer	Plos One	2014	88
6	Mager DL	The salivary microbiota as a diagnostic indicator of oral cancer: A descriptive, non-randomized study of cancer-free and oral squamous cell carcinoma subjects	Journal of Translational Medicine	2005	85
7	Castellarin M	*Fusobacterium nucleatum* infection is prevalent in human colorectal carcinoma	Genome Research	2012	80
8	Rubinstein MR	*Fusobacterium nucleatum* promotes colorectal carcinogenesis by modulating E-cadherin/β-catenin signaling via its FadA adhesin	Cell Host & Microbe	2013	80
9	Pushalkar S	Comparison of oral microbiota in tumor and non-tumor tissues of patients with oral squamous cell carcinoma	BMC Microbiology	2012	78
10	Kostic AD	*Fusobacterium nucleatum* Potentiates Intestinal Tumorigenesis and Modulates the Tumor-Immune Microenvironment	Cell Host & Microbe	2013	77

## Discussion

In this bibliometric analysis, we conducted a comprehensive evaluation and visualization of research on oral microbiota and cancer. Our analysis included a total of 1516 publications from the WoSCC database, covering the period from January 1, 2013, to December 31, 2022. Comparing this number to the publications by Liao Ga (2225 articles) [38] and Li Zhengrui et al. (3024 articles) [39], it is evident that a significant portion of the oral microbiota literature focuses on cancer. Our study also revealed a consistent increase in the number of publications in this field over time, underscoring the growing importance of understanding the role of oral microbiota in maintaining host health and contributing to disease development. Furthermore, the noninvasive, convenient, and rapid sampling method of the oral microbiota holds great promise as a diagnostic and prognostic biomarker for various diseases, attracting considerable attention from researchers [10].

Based on an analysis of countries, the USA and China have published the most publications in this field, which aligns with the findings of Liao Ga and Li Zhengrui et al. [38, 39]. Furthermore, five out of the top 10 institutions are from the USA, indicating a significant contribution of the country to the research on oral microbiota and cancer. It is worth noting that Zhou Xuedong’s team primarily focuses on the relationship between oral microbiota and both oral diseases and systemic diseases. The network maps depicted in Figs. 2, 3, and 5 reveal a growing interest among experts in the fields of oral microbiota and cancer, as well as active research and a global trend of academic communication, resource sharing, and collaboration among different countries, institutions, and researchers. This situation further promotes the progress of research in this field. Among the journals, *Frontiers in Cellular and Infection Microbiology* has published the highest number of papers, while *Plos One* is the most cited journal. Both of these journals have a profound impact on the field of oral microbiota and cancer.

Keywords co-occurrence analysis can provide insights into the prominent areas of research in the field of oral microbiota and cancer. Among the top 20 high-frequency keywords, three main research focuses were identified: oral microbiota (specifically *F. nucleatum*, *P. gingivalis*, and *Helicobacter pylori*), different types of cancers (such as colorectal cancer, squamous cell carcinoma, and oral cancer), and the underlying mechanisms (inflammation and infection). These findings suggest a correlation between chronic inflammation and cancer driven by oral bacteria. These results align with the study conducted by Liao Ga et al., who also identified periodontal disease, oral microbes, and squamous cell carcinoma as the prominent research areas [38].

*F. nucleatum* and *P. gingivalis* are two periodontal pathogens that have been found to play a significant role in oral, colorectal, and pancreatic cancer [1, 42]. For instance, a study by Okuyama et al. demonstrated that *P. gingivalis*, *F. nucleatum*, and *Prevotella intermedia* in the microenvironment produce lipopolysaccharide and secrete cytokines and molecules linked to carcinogenesis, tumor progression, invasion, and metastasis [43]. *F. nucleatum* is a Gram-negative anaerobic bacillus commonly found in the oral cavity, gastrointestinal tract, and other areas, and it is associated with various human diseases such as periodontitis, pregnancy outcomes, gastrointestinal disorders, and cardiovascular disease [44–47]. It is intriguing why *F. nucleatum* is connected to colorectal cancer. Since saliva mostly enters the gastrointestinal tract, there is a high likelihood of oral microbiota colonizing the intestinal tract, potentially influencing the development of the intestinal microbial community structure to some extent [48]. Additionally, the *F. nucleatum* found in colorectal cancer was genetically similar to strains of this species isolated from the mouth, suggesting that the *F. nucleatum* within the tumor may have originated from the oral cavity [8, 47]. Another possible pathway is that *F. nucleatum* present in the bloodstream could localize to colorectal cancer tissues and increase the risk of colorectal cancer [49]. Furthermore, numerous studies have identified a correlation between the presence of intratumoral *F. nucleatum* and worse survival outcomesl [50–52].

The relationship between oral microbiota and carcinoma development is complex and difficult to determine. However, oral microbiota can contribute to carcinogenesis through multiple mechanisms, such as inflammation, affecting proliferation/apoptosis, angiogenesis, and oncogene activation [1]. Inflammation plays a potential role in the initiation and progression of malignancy [8]. *F. nucleatum* can increase the infectivity of other pathogens, recruit tumor-infiltrating immune cells, activate NF-κB, cause the expression of pro-inflammatory cytokines (IL-1β, IL-6, IL-8, and TNFα) and antimicrobial peptide β-defensin 2. This creates a pro-inflammatory microenvironment that promote oral/colorectal tumorigenesis and progression [53–56]. Additionally, FadA serves as the direct bridge between *F. nucleatum* and cancer. It binds with E-cadherin, activating β-catenin signaling, subsequently enhancing the transcriptional activity of Wnt target genes, activation of pro-inflammatory cytokines, IL-6-STAT3 axis, oncogenic phenotype, and stimulation of the proliferation of cancer cells [57]. Furthermore, *F. nucleatum* Fap2 binds to Gal-GalNAc of colorectal cancer, engages TIGIT (an inhibitory immune receptor) on NK and T cells, and protects tumors from host immunity attack [58].

*P. gingivalis* is a Gram-negative anaerobic bacterium commonly found in the oral cavity. It has been linked to an increased risk of developing oral and digestive cancers through various mechanisms. These mechanisms include the production of carcinogenic substances, enhanced angiogenesis, induction of an inflammatory microenvironment, promotion of cell proliferation and invasion, and facilitation of epithelial-mesenchymal transition in malignant cells [59–61]. The activation of the PI3K/Akt and MAPK/ERK signaling pathways by *P. gingivalis* via gingipains has been found to significantly increase the percentage of S phase cells in the cell cycle and promote colorectal cancer cell proliferation [62]. Additionally, this bacterium promotes epithelial-mesenchymal transition by downregulating the expression of GSK3-beta and E-cadherin, while increasing pro-MMP9. It also stimulates tumor growth and metastasis by inhibiting p53 [63]. These findings not only provide valuable insights into cell biology research, but also contribute to a comprehensive understanding of the relationship between oral microbiota and cancer. They offer references for the development of precise targeting strategies and drugs against microorganisms, with the ultimate goal of inhibiting tumorigenesis and cancer progression.

Through our analysis of highly co-cited papers, we have identified the most important references in the field of oral microbiota and cancer. Table 6 shows that the advent of high-throughput sequencing has enabled the use of bacterial 16S rRNA gene sequencing, specifically the 16S V3-V4 region, as a powerful tool for exploring potentially oncogenic microbial composition species in various environments and human body districts, including the intestinal, oral, skin, and vaginal microbiota. These detection techniques have greatly contributed to the study of tumor microbiota [64]. Our results are consistent with Li Zhengrui et al., who also demonstrated that microbial sequencing technology has emerged as a significant topic in the field of oral microbiota [38]. Other detection methods for studying tumor microbiota include Fluorescence in Situ Hybridization, terminal restriction fragment length polymorphism, and denaturing gradient gel electrophoresis. The establishment of the Human Oral Microbiome Database (HOMD) has facilitated a better understanding of the oral microbiota [65, 66]. Research has revealed differential abundance of several oral microbiota taxa in oral cancer. For instance, the abundance of *Firmicutes* (*Streptococcus*) and *Actinobacteria* (*Rothia*) was significantly decreased, while *Capnocytophaga gingivalis*, *Prevotella melaninogenica*, and *Streptococcus mitis* were elevated. Other studies have shown a correlation between oral cancer and *Streptococcus sp.*, *Peptostreptococcus sp.*, *Prevotella sp.*, *Fusobacterium sp.*, *P. gingivalis*, and *Capnocytophaga gingivalis*. Furthermore, the oral microbiome can be utilized as a screening method for detecting oral cancer, achieving an 80% sensitivity and 83% specificity in a classification model [41, 67, 68]. These findings suggest that cancers can impact the abundance of oral microbiota, and that differences in oral microbiota could be utilized for risk prediction, diagnosis, treatment evaluation, and prognosis of cancers.

While it has been observed that cancer leads to changes in oral microbial diversity, the exact reasons behind these changes in the microbial community within the tumor microenvironment are not yet fully understood. It remains unclear whether these shifts occur because certain bacteria are better suited to adhere and grow in the tumor microenvironment or if they actively promote cancer. Exploring this direction could potentially become an emerging topic in the field of oral microbiota [67]. The composition of the tumor microbiome varies among different types of tumors, presenting a new opportunity to enhance our understanding of cancer pathogenesis [69, 70]. Moreover, it encourages interdisciplinary collaboration among dentists, oncologists, and oncologists to offer novel options for cancer patients [71].

The bibliometric analysis has certain inherent limitations. Firstly, all publications containing oral microbiota and cancer in the article topic were included in this study, ensuring the accuracy of the analysis results. However, it should be noted that this study only retrieved publications from the WoSCC databases and did not consider publications from other databases such as Scopus, Embase, Google Scholar, and PubMed. Future studies could benefit from including publications from these databases as well. Additionally, as the field continues to progress, it may be beneficial to involve experts in statistics and computer science to expedite the data extraction process, enhance the adequacy of result mining, and provide more accurate professional interpretations. Despite these limitations, we believe that the findings of this study offer a reliable and comprehensive understanding of oral microbiota and cancer research.

Overall, our study focuses on providing researchers with a comprehensive landscape of oral microbiota and cancer research. The increasing number of publications in this area indicates a growing understanding of the mechanisms underlying tumorigenesis. Our analysis highlights that the USA and China have contributed the most publications in this field. The keywords identified in this article suggest that the current hotspots revolve around studying the diversity of oral microbiota influenced by cancer and investigating the underlying mechanisms. These mechanisms could potentially guide the development of targeted therapies for tumorigenesis and serve as a reference for future research in this area.

### Supplementary Information

Additional file1 (DOCX 1848 KB)

## Data Availability

Research data are available from the corresponding author upon reasonable request.

## Abbreviations

WoSCC: Web of Science Core Collection database
IF: Impact factors
JCR: Journal citation reports
