# Supplementary material for: A bibliometric and visualized in oral microbiota and cancer research from 2013 to 2022
Source: Discov Oncol. 2024 Feb 1;15:24. doi: 10.1007/s12672-024-00878-5 (PMC10834930; doi:10.1007/s12672-024-00878-5)
Supplement: Supplementary file 1 — Additional file1 (DOCX 1848 KB) [file 12672_2024_878_MOESM1_ESM.docx]

**Material and methods**

Search strategy:

1# ((((((TS=(Tumor)) OR TS=(Neoplasm)) OR TS=(Neoplasia)) OR TS=(carcionoma)) OR TS=(Cancer)) OR TS=(Malignant Neoplasm)) OR TS=(Malignancy)

2# (((((((((((((((((((((((((TS=(oral microbiota)) OR TS=(oral microbiome)) OR TS=(oral microorganism)) OR TS=(oral microflora)) OR TS=(oral microbial community)) OR TS=(salivary microorganism)) OR TS=(saliva microbiota)) OR TS=(saliva microbiome)) OR TS=(salivary microflora)) OR TS=(salivarymicrobial community)) OR TS=(subgingival plaque microbiota)) OR TS=(subgingival plaque microorganism )) OR TS=(supragingival plaque microbiota)) OR TS=(supragingival plaque microorganism)) OR TS=(Mucosal Microbiome)) OR TS=(Mucosal microbiota)) OR TS=(Mucosal microorganism)) OR TS=(dental plaque)) OR TS=(dental microbiome)) OR TS=(dental microbiota)) OR TS=(dental microflora)) OR TS=(dental microbial community)) OR TS=(mouth microbiome)) OR TS=(mouth microbiota)) OR TS=(mouth microflora)) OR TS=(mouth microbial community)

3# (((TS=(gut microbiota)) OR TS=(gut microbiome)) OR TS=(gut microorganism)) OR TS=(gut microbial community)

4# 1# AND 2#

5# 4# NOT 3#

**Results**
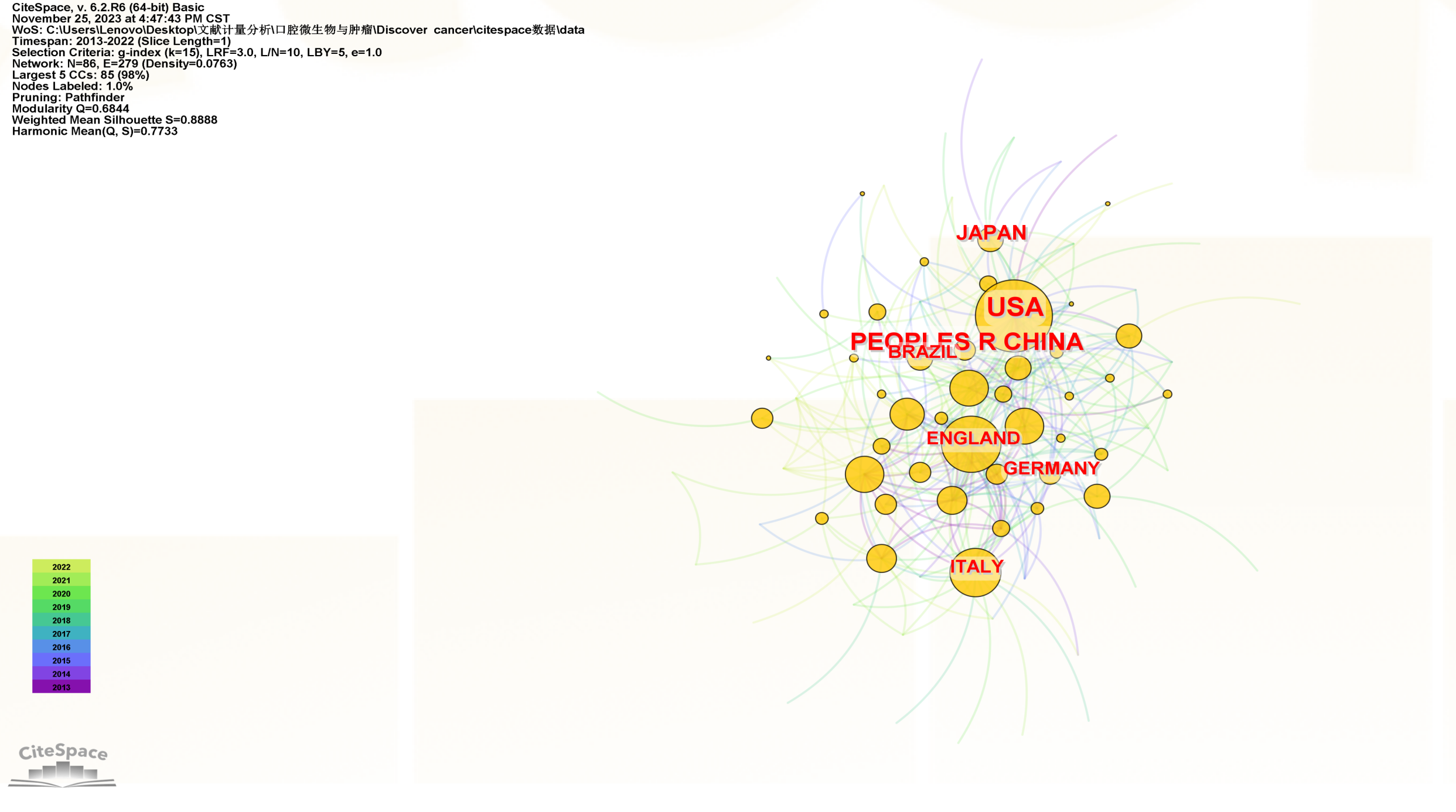


**Fig. S1** The network of countries in oral microbiota and cancer research


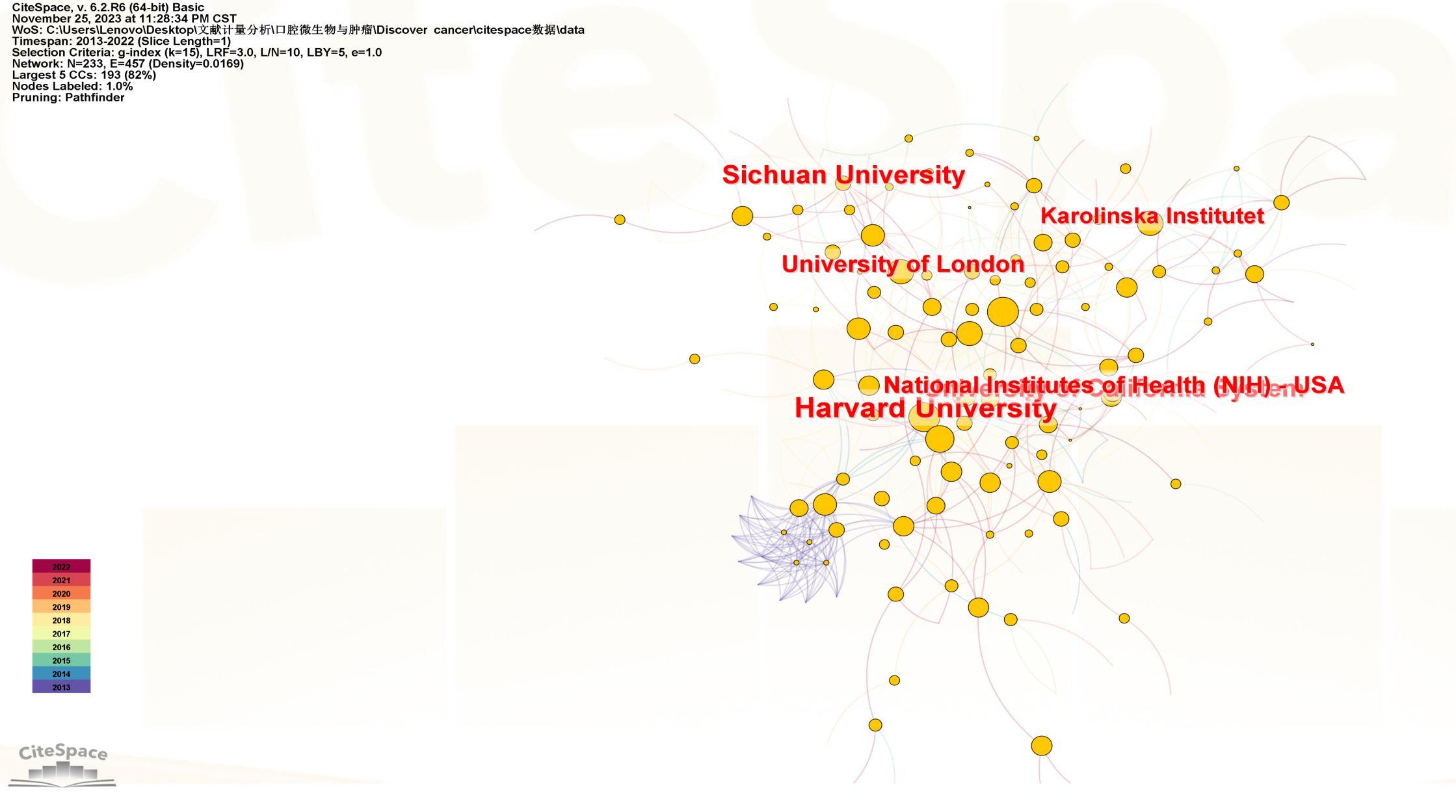


**Fig. S2** The network of institutions in oral microbiota and cancer research


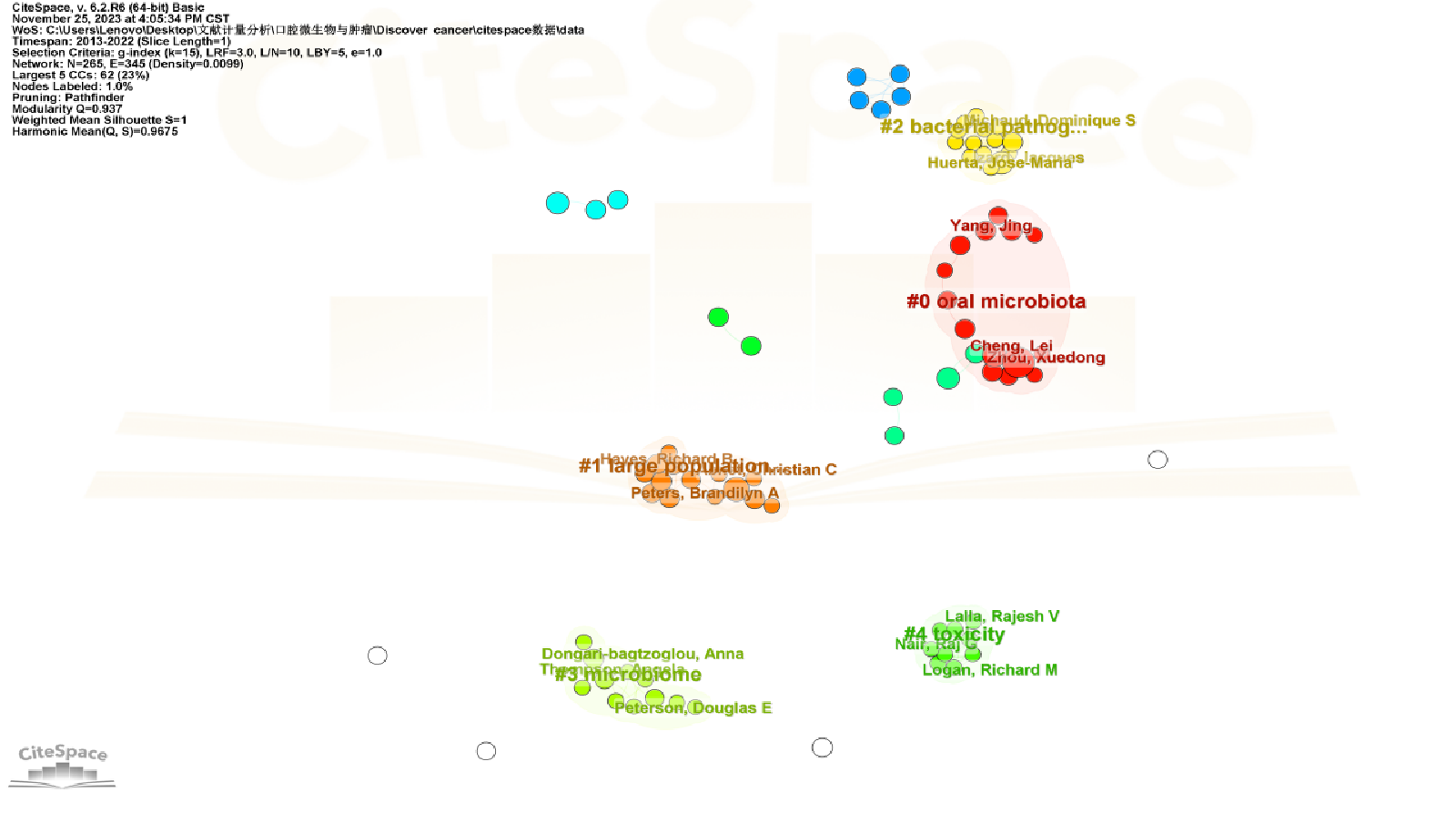


**Fig. S3** The network of authors in oral microbiota and cancer research
